# Recent Advances in Oral Drug Delivery Systems for BCS III Drugs

**DOI:** 10.3390/cimb48010063

**Published:** 2026-01-05

**Authors:** Junpeng Yu, Peng Wang, Zishen Bei, Lulu Tan, Jiaxin Wang, Guimin Qin, Yuying Huang, Shuhua Peng, Shen Liu, Jielan Huang, Xiaoxiu Shi, Qiujie Chen, Jinyan Xian, Yuanle Shen, Ting Xia, Jianfang Feng

**Affiliations:** 1 Institute of Traditional Chinese Medicine Zhuang-Yao Medicine Research, Guangxi University of Chinese Medicine, Nanning 530200, China; y3320682971@163.com (J.Y.); wang2758336312@163.com (P.W.); 13417203095@163.com (Z.B.); tll15777599412@163.com (L.T.); 18836935210@163.com (J.W.); 18290066248@163.com (G.Q.); hyy0242@163.com (Y.H.); 15807801326@163.com (S.P.); 13878835241@163.com (S.L.); huangjielan3507@163.com (J.H.); 18878634471@163.com (X.S.); 18376038045@163.com (Q.C.); xianjy2024@163.com (J.X.); shenyuanle2022@163.com (Y.S.); 2 Guangxi Engineering Technology Research Center of Advantage Chinese Patent Drug and Ethnic Drug Development, Guangxi University of Chinese Medicine, Nanning 530200, China

**Keywords:** BCS III drugs, oral drug delivery, intestinal permeability barrier, bioavailability enhancement

## Abstract

Oral drugs classified under Class III of the Biopharmaceutics Classification System (BCS) are defined by high aqueous solubility yet low intestinal permeability. Their restricted oral bioavailability arises not from inadequate dissolution, but is primarily governed by the intestinal permeability barrier, coupled with substantial inter-individual variability in absorption. This review adopts the intestinal permeability barrier as its core analytical framework to dissect the key determinants of oral absorption for BCS III drugs, while presenting a comparative and critical evaluation of prevailing bioavailability enhancement strategies. From perspectives including mechanism of action, achievable magnitude of enhancement, applicable physicochemical and physiological conditions, and translational feasibility, the intrinsic mechanistic limitations and applicable boundaries of distinct strategies are delineated. Finally, this paper concludes that the absorption barriers of BCS III drugs cannot be universally surmounted by a single strategy, emphasizing the significance of mechanism-guided strategy selection for the rational design of oral drug delivery systems. In doing so, it provides a foundational basis for the rational development of oral delivery systems tailored to BCS III drugs.

## 1. Introduction

Oral administration, the most predominant route of drug delivery in clinical practice, accounts for approximately 80–90% of globally marketed pharmaceutical dosage forms, and its long-standing dominance is attributed to its favorable safety profile, convenience, and high patient compliance [1]. According to statistics from the U.S. Food and Drug Administration (FDA) and the World Health Organization (WHO), approximately 25–40% of marketed oral drugs are classified as BCS III. The BCS classification framework is detailed in Table 1. BCS III drugs typically exhibit high aqueous solubility; however, their high polarity and low lipophilicity result in poor intestinal permeability, which compromises drug absorption and bioavailability, thereby limiting their clinical utility. Representative BCS III agents include metformin, atenolol, and acyclovir—these drugs hold pivotal roles in the management of chronic conditions such as diabetes mellitus, hypertension, cardiovascular diseases, viral infections, and infectious diseases, and serve as cornerstones of long-term pharmacotherapy for patients [2]. Consequently, despite their well-characterized pharmacology, the clinical performance and developability of BCS III drugs remain constrained by their intrinsic physicochemical properties.

Currently, enhancing intestinal permeability stands as one of the primary challenges in the development of BCS III drugs. Owing to permeability barriers, BCS III drugs struggle to traverse the lipid bilayer of intestinal epithelial cells, leading to low oral absorption efficiency. Additionally, physiological factors—including gastrointestinal pH, prandial state, intestinal transporter activity, and substantial inter-individual variability—further exacerbate the uncertainty of drug absorption, collectively limiting their widespread clinical application [3].

To address these challenges, researchers have proposed a range of strategies to enhance the bioavailability of BCS III drugs. For example, phospholipid complexation, self-emulsifying drug delivery systems (SEDDS), nanotechnology, and permeation enhancers have been extensively investigated. These approaches improve bioavailability by increasing drug solubility, enhancing intestinal permeability, or expanding the absorption surface area. However, while these methods enhance permeability, ensuring the safety, stability, and efficacy of the drugs remains a critical issue that needs to be addressed urgently [4].

## 2. Mechanisms of Oral Drug Absorption

The absorption of oral drugs is a complex multi-step process, which primarily encompasses several key steps including drug dissolution, traversal across the gastrointestinal barrier, and entry into the systemic circulation.

First, upon entering the gastrointestinal tract, the drug initiates dissolution. Solubility stands as one of the critical factors influencing drug absorption—only when the drug dissolves into appropriate ionic or molecular forms can it be absorbed by the intestinal tract [5]. For water-soluble drugs, the dissolution process typically proceeds rapidly. In contrast, lipophilic drugs may necessitate the aid of emulsification, solvents, or other excipients to achieve effective dissolution and access the absorption pathway [6].

Following complete dissolution, the drug must traverse the membrane of intestinal epithelial cells. The intestinal epithelial cell membrane, composed of a phospholipid bilayer, serves as the primary barrier to drug entry into the body. During the traversal of intestinal epithelial cells, drugs primarily rely on two mechanisms: passive diffusion and active transport [1]. Passive diffusion is applicable to small-molecule or highly lipophilic drugs. Driven by concentration gradients, these drug molecules can spontaneously permeate the intestinal epithelial cell membrane. In contrast, drugs with larger particle sizes or high polarity typically require transporters located on the intestinal epithelial cell membrane to facilitate their active transport from low-concentration regions into cells—this process, which consumes energy, enables the drugs to cross the intestinal epithelial barrier against the concentration gradient [7].

Beyond cell membrane penetration, drugs may also enter the systemic circulation via the paracellular pathway. For certain small-molecule drugs, they can readily traverse the tight junctions (TJs) between intestinal epithelial cells to access the systemic circulation [8]. However, given the narrow intercellular spaces, this pathway imposes inherent limitations on the absorption of macromolecular drugs.

Upon successful traversal of intestinal epithelial cells, the drug enters the bloodstream. The majority of drugs are transported to the liver via the portal venous system, where they undergo metabolism prior to entering the systemic circulation. Certain lipophilic drugs may bypass the first-pass effect through the lymphatic system, directly accessing the bloodstream; this pathway circumvents the impact of hepatic metabolism, thereby enhancing the drug’s bioavailability [9].

## 3. Factors Governing the Absorption of BCS III Drugs

### 3.1. Membrane Permeability Barrier

Membrane permeability barrier is the primary determinant of bioavailability for BCS III drugs. In contrast to BCS II agents, the bioavailability of BCS III drugs is not solubility-limited, but rather predominantly governed by intestinal permeability [10]. Owing to their inherent possession of multiple hydrogen-bonding moieties, BCS III drugs typically exhibit high polarity; additionally, these agents often have large molecular sizes and bear strong charges [11]. However, given that the upper intestinal epithelium is composed of lipid bilayers, this structure conversely impedes the absorption of BCS III drugs [12]. Consequently, low permeability constitutes an intrinsic limitation that restricts the capacity of BCS III drugs to translocate from the intestinal lumen into the systemic circulation, leading to poor oral bioavailability. For example, ganciclovir, a BCS III drug, exhibits an oral bioavailability of merely ~5%; this is attributed to its large molecular size and possession of polar moieties, which impede its intestinal absorption [13].

The ionization state of BCS III drugs also exerts a substantial influence on their absorption. In general, un-ionized drug species exhibit greater lipophilicity, thereby facilitating their permeation across biological membranes. However, many BCS III drugs are inherently highly hydrophilic and undergo extensive ionization in the gastrointestinal tract; this further diminishes their lipophilicity, rendering passive diffusion even more unfavorable [14]. Furthermore, fluctuations in the pH of the small intestine can modulate the degree of ionization of weak acidic or weak basic drugs, resulting in certain drugs predominantly existing in their ionized form—a state that further diminishes their permeability [15]. Thus, despite the favorable solubility of BCS III drugs in the gastrointestinal milieu, their ionization properties still give rise to permeability barriers.

Furthermore, transporters localized on the surface of intestinal epithelial cells modulate the absorption of BCS III drugs. On one hand, efflux transporters mediate the energy-dependent directional efflux of drugs that have entered epithelial cells back into the intestinal lumen, thereby significantly reducing the net flux of transmembranous drug transport. On the other hand, uptake transporters facilitate carrier-mediated transmembranous transport, providing an alternative transcellular pathway for certain hydrophilic drugs and thus partially offsetting their limited capacity for passive diffusion [16]. For instance, P-glycoprotein (P-gp) and BCRP limit drug absorption via efflux mechanisms, whereas PEPT1, OATP, and SMVT enhance drug absorption and thereby improve bioavailability. Conversely, in the absence of such transporters or when transporter expression is low, drug permeability is significantly diminished. For example, valacyclovir is absorbed via the PEPT1 transporter in the proximal small intestine, resulting in high bioavailability [17]. Yang et al. demonstrated that the transport rate of gliclazide is reduced in the variant OATP1B1 transporter, indicating that its absorption is significantly constrained by transporter function—particularly in individuals with low transporter expression or impaired transporter activity [18]. Furthermore, numerous BCS III drugs may also act as substrates for transporters. For example, fexofenadine, a substrate of P-gp, has its intestinal absorption limited by efflux transporters—particularly in small intestinal segments with relatively high P-gp expression, which impairs the effective absorption of the drug [19].

On this basis, the permeability profile of a drug is also closely associated with its dosage and the saturation status of transporters [20]. At higher doses, transporters may reach saturation; under such circumstances, even if the drug is dependent on transporters for absorption, the absorbed amount may not increase proportionally with escalating doses [21]. Under such circumstances, the oral bioavailability of drugs often exhibits dose-dependent or disproportionate alterations—a phenomenon particularly prevalent among BCS III drugs. For example, Sorzano validated via in vitro assays and rat intestinal perfusion experiments that high drug doses may induce transporter saturation, resulting in non-proportional increases in drug absorption with escalating doses and thus eliciting dose-dependent changes in bioavailability; this effect is especially prominent for BCS III drugs [22].

### 3.2. Paracellular Pathway Restrictions

In scenarios where membrane permeability is rate-limiting, BCS III drugs can theoretically access the systemic circulation via the paracellular pathway—i.e., traversing the intercellular spaces between intestinal epithelial cells. However, under normal physiological conditions, the flux of this pathway is inherently tightly constrained. This constraint arises from the highly occlusive TJs of intestinal epithelial cells, which not only preserve the integrity of the epithelial barrier but also impede the permeation of harmful substances [23]. TJs not only dictate the permeability of intercellular spaces but also exhibit distinct size selectivity and charge selectivity, typically permitting the passage of only a limited number of small hydrophilic molecules [24].

However, owing to their large molecular weight, high charge density, or complex spatial conformation, BCS III drugs—even when fully solubilized in the intestinal lumen—exhibit poor permeability across TJs, leading to suboptimal overall absorption. Furthermore, the permeability of TJs varies across distinct intestinal segments and is modulated by inflammatory states, dietary factors, and cytoskeletal regulation, among other factors, which further exacerbates the instability and inter-individual variability in the absorption of BCS III drugs [25]. For instance, studies by Capaldo et al. have demonstrated that the paracellular permeability of TJs is not static; instead, it is dictated by the combinatorial expression of distinct claudin subtypes, thereby conferring pronounced size and charge selectivity for solute passage. Furthermore, claudin expression exhibits regional heterogeneity across different intestinal segments and can be dynamically regulated by pro-inflammatory factors under conditions such as inflammation—events that induce fluctuations in barrier permeability and further exacerbate inter-individual variability in drug absorption [26].

### 3.3. Drug Stability Factors

Drug stability factors also constitute a critical determinant of the oral bioavailability of BCS III drugs [27]. For BCS III drugs, the permeability of the small intestine largely constrains their oral absorption efficacy; consequently, alterations in the gastrointestinal environment exacerbate the challenges arising from their permeability deficiency [28].

Following food intake, elevated gastric pH and delayed gastric emptying not only prolong the transit time of drugs into the small intestine but also may result in drug release being initiated exclusively in the distal small intestine, thereby diminishing the effective absorption surface area [29]. Furthermore, food components may form a coating around the pharmaceutical formulation, thereby retarding the rate of drug disintegration and dissolution [30].

High-fat diets, in particular, markedly increase the viscosity of gastrointestinal contents, retard drug release, and may induce a negative food effect [31]. Thus, the formulation design of BCS III drugs should optimize the composition to reduce sensitivity to variations in the viscosity of gastrointestinal contents, thereby mitigating the adverse effects of food on drug absorption [32].

### 3.4. Other Factors

Beyond the primary barriers outlined above, additional physiological and pharmacokinetic factors may also modulate the oral absorption of BCS III drugs. For example, the first-pass effect can result in extensive drug metabolism prior to systemic circulation entry, leading to a substantial reduction in plasma drug concentration. Furthermore, variations in gastric emptying rate, small intestinal transit time, and luminal fluid volume may all influence the residence time and effective concentration of BCS III drugs at the absorption site. Specifically, accelerated small intestinal propulsion or a marked increase in intestinal fluid volume can substantially reduce the contact time of the drug within the effective absorption window, thereby limiting the extent of its oral absorption [31].

**Figure 1:**
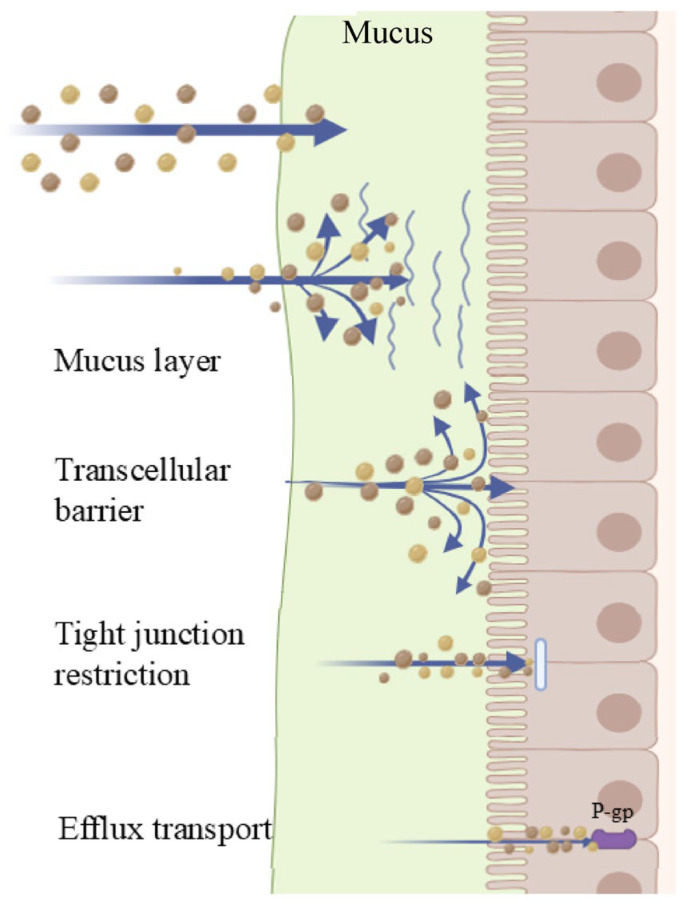
Major intestinal permeability barriers limiting the oral absorption of BCS III drugs.

Gastrointestinal physiological disparities across different age groups may also modulate the absorption of BCS III drugs. For example, children, the elderly, or patients with compromised gastrointestinal function exhibit more pronounced differences in gastrointestinal conditions compared to healthy adults, rendering these populations more susceptible to variations in drug absorption and leading to greater inter-individual variability in bioavailability [33,34]. Thus, in the formulation design and clinical application of these drugs, the physiological disparities of special populations should be fully taken into account. The major intestinal permeability barriers limiting the oral absorption of BCS III drugs are illustrated in Figure 1.

### 3.5. Molecular and Biochemical Regulation of Intestinal Barriers

Beyond the physical and biological barriers discussed earlier, the intestinal permeability to BCS III drugs is dynamically regulated by intracellular signaling pathways. This is attributed to the fact that TJs are not static seals; the opening and closure of their pores are intricately linked to complex signaling cascades [35]. For example, the myosin light chain kinase pathway and Rho kinase signaling represent core mechanisms governing paracellular permeability. These pathways can induce the phosphorylation of tight junction proteins (e.g., occludin and claudins), which in turn triggers contraction of the peripheral actomyosin ring [36]. Brunner and others have proposed and experimentally validated a new class of tight junction modulators. By targeting and binding to cell membrane receptors or tight junction proteins, they can activate intracellular signaling cascades such as MLCK, thereby inducing reversible contraction of the peripheral actin-myosin ring to temporarily open the channels [24]. This biochemical regulation strategy rooted in molecular recognition has successfully advanced formulation development beyond the mere overcoming of physical barriers, shifting its focus toward the modulation of the physiological state of intestinal epithelial cells.

Furthermore, the absorption efficacy of BCS III drugs is intimately linked to the molecular biological properties and genetic background of their transporters. Notably, the expression levels and functional activities of membrane transporters such as P-glycoprotein (P-gp, encoded by ABCB1) and peptide transporter 1 (PEPT1, encoded by SLC15A1) exhibit substantial inter-individual variability, which is primarily driven by genetic polymorphisms [37]. Genetic heterogeneity not only dictates the transmembrane capacity of drugs within the intestinal epithelium but also contributes to substantial inter-individual variability in the clinical performance of pharmaceutical formulations [38]. For example, through clinical trials conducted on healthy subjects, Saiz-Rodríguez et al. demonstrated that single-nucleotide polymorphisms (SNPs) in transporters including OCT1, OCT2, and MATE2 significantly alter the pharmacokinetic profiles of metformin—a prototypical BCS III drug. This finding confirms that genetic variations, by modulating the molecular functions of transporters, directly contribute to marked inter-individual differences in drug absorption and clearance rates [39].

Finally, certain excipients can directly disrupt cellular energy homeostasis. For example, they may induce intracellular ATP depletion by interfering with the mitochondrial respiratory chain, thereby abrogating the energy supply for ATP-dependent efflux pumps [40]. This intervention effectively inhibits the transport activity of efflux pumps. By reducing the transmembrane resistance of BCS III drugs, it significantly enhances their oral absorption efficiency and bioavailability. Thus, future drug delivery strategies should place greater emphasis on matching the expression profiles and molecular regulatory mechanisms of transporters, thereby overcoming absorption barriers arising from biological heterogeneity. Collectively, these physiological, molecular, and genetic factors define the applicability boundaries of absorption-enhancement strategies for BCS III drugs, indicating that their effectiveness is inherently context- and drug-dependent.

## 4. Strategies for Enhancing Absorption

**Figure 2:**
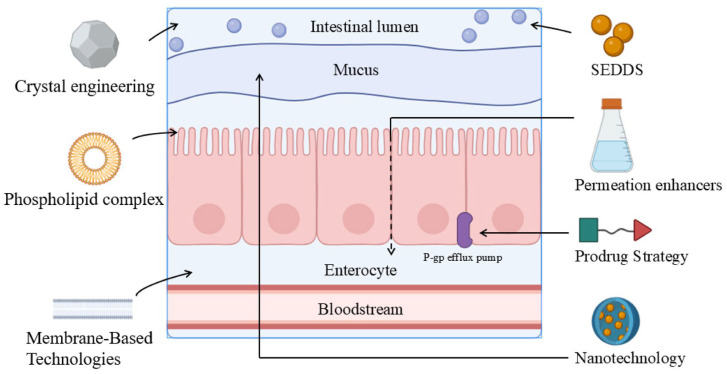
Intestinal absorption barriers and intervention sites of major strategies for BCS III drugs.

As elaborated in Section 3, the core limitation of oral absorption for BCS III drugs does not stem from insufficient dissolution, but rather arises primarily from the combined effects of multi-level permeability-associated barriers. These include the transcellular membrane permeability barrier, restricted paracellular pathways, drug stability-related impacts, and other factors such as food effects and gastrointestinal physiological disparities. In response to these constraints, researchers have proposed targeted countermeasures: For the transcellular membrane permeability barrier (Section 3.1), absorption enhancement is typically achieved by increasing the drug’s apparent lipophilicity and membrane partitioning capacity, as well as augmenting the driving force for transmembrane permeation—representative strategies encompass phospholipid complexes, chemical modification, and crystal engineering. For paracellular pathway restrictions (Section 3.2), the key approach involves modulating tight junction-associated permeability in a controllable and reversible manner to promote paracellular transport, with permeation enhancers being the most commonly employed strategy. For drug stability factors (Section 3.3), the focus lies in protecting the drug and sustaining effective concentrations within the complex gastrointestinal environment via encapsulation and similar techniques, where nano-delivery systems are frequently utilized. For other factors (Section 3.4), fluctuations are mitigated primarily by improving the consistency of in vivo exposure and enabling spatiotemporal control over drug release—SEDDS and membrane technologies serve as representative strategies. The subsequent sections will systematically review and discuss the mechanisms of action and applicability of the aforementioned strategies. The main absorption barriers of BCS III drugs and the implementation sites of intervention measures are shown in Figure 2.

### 4.1. Phospholipid Complexation Strategy

The phospholipid complexation strategy refers to a technique that conjugates drugs with phospholipids to form stable complexes. This complexation approach can effectively prevent drugs from being degraded by metabolic enzymes. Owing to its favorable biocompatibility and non-toxicity, the phospholipid complexation strategy has been widely employed in oral pharmaceutical formulations [41]. This strategy primarily enhances drug solubility via the emulsifying action of phospholipids and mimics the natural lipid absorption mechanism to form a cell membrane-mimetic structure. While augmenting membrane permeability, it facilitates the translocation of drugs across intestinal cell membranes into the systemic circulation with greater ease [42].

**Figure 3:**
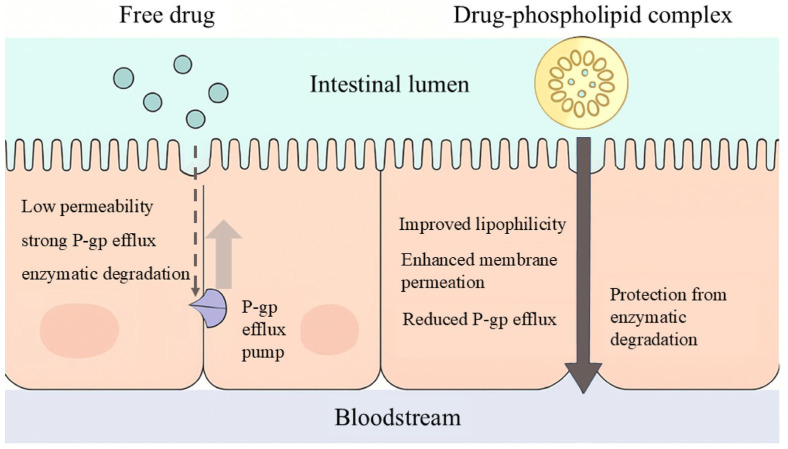
Schematic diagram of the phospholipid complexation strategy mechanism.

Beyond active transport, phospholipid complexes can effectively mitigate the impact of intestinal P-gp-mediated efflux by sequestering polar hydrogen bond donors and acceptors. A schematic illustration of the mechanism is provided in Figure 3. For example, Hashemzadeh et al. demonstrated that upon conjugation of polyphenols with phosphatidylcholine, phosphatidylcholine engages in the self-assembly of the complex via its long hydrophobic chains, thereby forming a hydrophobic layer surrounding the polyphenol molecules [43]. Mandeep conjugated FEX, a prototypical BCS III drug, with soy-derived Phospholipon^®^ 90H via the solvent evaporation method to form the FEX-phospholipid complex. Notably, the P-gp-mediated efflux ratio of FEX-phospholipid complex was reduced from 4.04 (for free FEX) to 1.34 [44]. Qin et al. enhanced the oral bioavailability of bergenin via phospholipid complex technology, with the area under the plasma concentration-time curve (AUC) and maximum plasma concentration (C_max_) increasing by approximately 4.39-fold and 4.46-fold, respectively [45]. Furthermore, Xia et al. enhanced the bioavailability of salvianolic acid B via phospholipid complexation, leading to an approximate 2.31-fold increase in its AUC. Moreover, by further incorporating a novel micelle-based composite system, the AUC was elevated to 3.40-fold relative to the free drug [46]. In addition, a variety of preparation techniques, including ultrasonication, freeze-drying, high-pressure homogenization, and solvent co-precipitation, have been employed for the complexation of diverse drug types with phospholipids, thereby offering multiple insights for this strategy.

Simple binary complexation can ameliorate the solubility limitations of drugs, whereas multi-component complexation is more conducive to leveraging the advantages of complexes. For example, Arafat et al. incorporated bile salts into liposomes; this modification not only enhanced the intestinal solubility of the drug cefotaxime via the liposomal carrier but also exerted the stabilizing effect of bile salts [47]. Fan et al. first complexed the poorly soluble drug baicalein with phospholipids to successfully enhance its lipophilicity, followed by combination with Soluplus. This multi-step modification significantly improved the flowability of the originally semi-solid binary complex [48].

Although the phospholipid complexation strategy demonstrates substantial potential for improving the bioavailability of BCS Class III drugs, its practical application is still constrained by multiple factors. First, the physicochemical properties of phospholipid complexes may lack sufficient stability and are prone to being affected by environmental factors, leading to the dissociation of the complexes or a reduction in their performance [49,50]. Second, the preparation processes of phospholipid complexes are relatively complex, requiring high-precision control, while exhibiting considerable batch-to-batch variations that may compromise the stability and reproducibility of production [51,52]. Furthermore, while the phospholipid complexation strategy exhibits excellent performance in enhancing drug solubility and membrane permeability, it does not confer benefits to all drugs. For example, in their research on the drug design of 20(S)-Protopanaxadiol, Pu et al. noted that due to the insufficient stability of phospholipid complexes, they are typically employed as intermediates in pharmaceutical formulations and require combination with other delivery systems to achieve more stable and effective oral administration outcomes [53]. It can be observed that for drugs with high hydrophilicity or complex structures, the modification efficacy of phospholipid complexes may not meet expectations, or such complexes may need to rely on other technologies (e.g., nanotechnology or permeation enhancers) to further improve the stability and bioavailability of the drugs [54].

Thus, the phospholipid complexation strategy represents an effective approach to enhancing drug bioavailability, particularly for pharmaceuticals primarily limited by passive transcellular permeability. By improving the compatibility between drugs and biological membranes, it boosts absorption efficiency without modifying the drugs’ covalent structures—rendering it more suitable for agents with moderate lipophilic potential. However, in practical applications, this strategy still confronts challenges including inadequate stability, process complexity, and insufficient characterization methodologies. Future research should prioritize enhancing the stability and applicability of the complexes via the introduction of novel materials, as well as improving production consistency through process optimization and standardization.

### 4.2. Prodrug

Beyond formulation-based phospholipid complexation, molecular-level optimization offers an alternative direct approach to enhancing the permeability of BCS III drugs. A prodrug is defined as a derivative generated via chemical modification of an active drug molecule, which undergoes a biologically reversible reaction in vivo to release the parent active drug [55,56]. For BCS III drugs, the prodrug strategy primarily acts by modifying the physicochemical properties of the drug, thereby enhancing its permeability [57].

This strategy enables the circumvention of in vivo drug absorption barriers without altering the pharmacological efficacy of the parent drug, while simultaneously enhancing its solubility, permeability, and bioavailability. Furthermore, prodrugs can improve the specific targeting of drugs, for example, via enzymatic conversion to release the active drug [58]. For example, Gabapentin enacarbil, a prodrug of Gabapentin, was modified via esterification by Lal et al. to enable active transport by multiple small intestinal transporters. This prodrug enhances the oral bioavailability of Gabapentin to over 68% and circumvents the inherent dose-dependent absorption saturation of Gabapentin by extending the absorption window and reducing absorption saturation [59,60]. C2E5, a prodrug of diethylenetriaminepentaacetic acid (DTPA), was subjected to esterification modification by Sadgrove et al. to enhance its lipophilicity, which in turn improved its permeability across the intestinal membrane. In animal models, the oral therapeutic efficacy of C2E5 was significantly superior to that of unesterified DTPA, with a marked increase in the removal efficiency of ^241^Am. Importantly, oral administration of C2E5 achieved efficacy comparable to that of intravenous Ca-DTPA [61]. Wang et al. increased the oral bioavailability of gabapentin to over 68% by designing an ester prodrug that could be actively transported by MCT1 and SMVT, effectively overcoming the limited bioavailability of the original drug due to low membrane permeability and absorption saturation [62].

In clinical settings, the primary risks associated with the prodrug strategy predominantly encompass interindividual variability in metabolic conversion, the potential formation of toxic metabolites, and reliance on physiological mechanisms [63]. For example, Lee et al. demonstrated that clopidogrel, a prodrug, requires metabolic activation via the hepatic CYP2C19 enzyme to generate its active metabolite. In individuals with low CYP2C19 gene expression levels, the production of the active metabolite is diminished, which reduces the therapeutic efficacy of the drug and elevates the risk of cardiovascular events [64].

Thus, in comparison to the phospholipid complexation strategy discussed in Section 4.1, the prodrug strategy not only improves physicochemical properties but also remodels the transmembrane transport mechanism of drugs at the molecular level. Nevertheless, during the prodrug modification process, more rigorous pharmacokinetic studies are required for evaluation to mitigate the risks associated with interindividual variability.

### 4.3. Crystal Engineering

Beyond chemical modification, crystal engineering serves as an alternative strategy to modulate interfacial driving forces and membrane partitioning without permanently altering the covalent structure of the drug.

Crystal engineering is a strategy that systematically modulates the physicochemical properties of solid-state drugs by designing and manipulating intermolecular non-covalent interactions to alter their crystal structures and lattice environments [65]. Synthons typically encompass homotypic and heterotypic hydrogen-bonded networks, and the formation of salts or cocrystals can be predicted based on the pKa difference between the ligand and the drug [66,67]. Crystal engineering is particularly well-suited for molecules that are recalcitrant to salt formation or for which altering the ionization state is inadvisable. Furthermore, as a solid-state modification strategy, it offers enhanced manufacturability and improved stability.

By modulating intermolecular interactions and the lattice environment within crystals, crystal engineering can generate a greater chemical potential difference at the absorption interface, thereby increasing the concentration gradient and accelerating diffusion. Additionally, the novel packing arrangement of cocrystals weakens lattice constraints, while the incorporation of lipophilic cocrystal ligands facilitates improved membrane partitioning of the drug, thereby enhancing transmembrane diffusion [68]. For example, the selection of highly water-soluble ligands can enhance apparent solubility, thereby increasing permeation flux via concentration gradients; conversely, conjugation with lipophilic ligands improves lipophilicity, which in turn strengthens membrane affinity [69].

For example, Zhao et al. formulated famotidine as a salt-cocrystal, which resulted in an approximately 1.09-fold increase in its aqueous solubility and a roughly 1.75-fold enhancement in the 8 h cumulative permeation across a biomimetic membrane. This phenomenon is attributed to the synergistic effect of the stronger chemical potential driving force exerted by the salt-cocrystal at the membrane interface and the improved membrane partitioning capacity conferred by the ligand’s higher logP value [70]. Furthermore, Li et al. fabricated a cocrystal of Biochanin A and nicotinamide, which led to a significant enhancement in its aqueous solubility and in vitro release rate. Following oral administration, the AUC was increased by approximately 2.1-fold, and the C_max_ was elevated by roughly 1.8-fold [71]. Dai et al. modulated the pharmacokinetic properties of 5-fluorouracil (5FU) through cocrystallization with dihydroxybenzoic acids; notably, the AUC of the 5FU-25DHBA cocrystal was approximately 2.78-fold that of the parent drug [72].

Despite the substantial advantages of crystal engineering in enhancing drug bioavailability, it is frequently associated with the risk of solution-mediated phase transformation (SMPT). SMPT is defined as a process wherein metastable solids initially form supersaturated solutions during dissolution, followed by nucleation and growth at the solution interface into more stable phases with lower solubility, thereby reducing the dissolution rate and attenuates the improvements in solubility and permeability conferred by cocrystals [73]. Thus, during the early development stage, concurrent monitoring of dissolution and phase transformation across diverse systems is imperative. Additionally, modulating conditions such as ionic strength to reduce the driving force for recrystallization, coupled with screening solid forms that remain phase-stable under target conditions based on suspension and medium stability assessments, enables the mitigation of SMPT risks at the source [74].

For example, Singh et al. proposed the simultaneous monitoring of dissolution and recovery of solid residues in parallel artificial membrane permeability assay, Franz diffusion cell, or Caco-2 permeability experiments. By means of powder X-ray diffraction and differential scanning calorimetry, they aimed to determine whether the system transitions from a supersaturated state to a stable phase. The risk of SMPT is identified if a concentration peak is followed by a decline and the stable phase is detected [75]; Chappa et al. demonstrated that entinostat can undergo transformation from its anhydrous form to a hydrate when suspended in specific media. Based on this observation, they selected the hydrate as the target solid form for pharmaceutical development, thereby mitigating the risk of recrystallization [76]. Chen et al. performed coupled dissolution and phase transition characterization in the target medium. Their findings revealed that in the absence of bile salts, SMPT occurred rapidly, with supersaturation being transient. In contrast, the addition of sodium taurocholate inhibited phase transition and significantly prolonged the supersaturation state. Consequently, sodium taurocholate was selected as the medium to mitigate the risk of SMPT [77].

Thus, analogous to the first two strategies, crystal engineering is equally applicable to addressing passive transcellular permeation barriers. By modulating interfacial behaviors to optimize the balance between dissolution and permeation, crystal engineering enables modification of drugs without altering their covalent structures. However, the risk of SMPT remains a substantial challenge for this approach. Therefore, in practical applications, researchers should make optimized selections within this technology cluster based on the chemical stability and crystallization kinetics of the target drug.

### 4.4. Penetration Enhancers

The low permeability of BCS III drugs constitutes the primary contributor to their poor bioavailability; accordingly, enhancing permeability has emerged as a pivotal strategy to facilitate transmembrane absorption. TJs—cell junctional complexes localized at the apical region of adjacent epithelial cells—maintain epithelial barrier integrity by occluding intercellular spaces, which precisely accounts for their role in impeding the permeation of BCS III drugs. Hence, modulating cytoskeletal architecture to transiently open TJs represents the core strategy for augmenting the permeability of BCS III drugs [23,24]. For example, Ashmawy et al. incorporated D-glucose and NaCl into oral formulations and observed that D-glucose activated the SGLT1 in the intestinal epithelium, thereby increasing water flux and inducing a pronounced solvent drag effect. This effect triggered transient dilation of TJs, which significantly enhanced the oral bioavailability of BCS III drugs, including ranitidine, atenolol, and acyclovir [78]. Building upon this foundation, Mady et al. demonstrated that natural honey exhibits a pronounced permeation-enhancing effect. Notably, when compared to equimolar D-glucose, honey more effectively induced the dilation of intercellular TJs, resulting in a 10.09-fold increase in the oral bioavailability of acyclovir. Thus confirming honey as a highly promising natural permeation enhancer [79].

Second, modulating cell membrane architecture via surfactants represents an alternative strategy for enhancing drug permeation. Surfactants can reduce intercellular electrical resistance and disrupt cell membrane integrity, thereby augmenting the accumulation of drugs in target tissues [23]. For example, Deshmukh et al. demonstrated that the nonionic surfactant dodecyl maltoside can reduce transepithelial electrical resistance, thereby facilitating the transmembrane transport of BCS III drugs, including tiludronate and cromolyn [80].

While permeability enhancers can improve the intestinal permeability of BCS III drugs via the modulation of TJs, this strategy is inherently limited at the mechanistic level. As a structural component responsible for preserving the integrity of the intestinal epithelial barrier, TJs do not solely function in regulating drug translocation; rather, they exert a barrier effect against a broad spectrum of exogenous substances [81]. Thus, the permeation enhancement strategy that relies on TJs opening as its core mechanism is frequently associated with the risk of global impairment of the intestinal epithelial barrier function [82]. For instance, studies conducted by Metry and Polli et al. have revealed that while sugar alcohol-based permeation enhancers can modulate paracellular transport, high concentrations induce osmotic effects that significantly shorten intestinal transit time—conversely reducing bioavailability due to insufficient drug residence time. Additionally, surfactants, at concentrations required for permeation enhancement, tend to elicit cytotoxicity and non-selective disruption of barrier function, thereby elevating the risk of endogenous toxin invasion [83].

Modulation of cell membrane architecture via surfactants can similarly reduce transepithelial electrical resistance and enhance drug flux; however, the effective dose window for this strategy is generally narrow [84]. Excessive exposure may trigger irreversible membrane damage or epithelial inflammatory responses. This risk cannot be fully mitigated solely through precise dose titration; instead, it arises from the mechanism of direct interference with membrane integrity. For example, studies by Maher et al. have demonstrated that surfactants such as sodium dodecyl sulfate—while significantly enhancing the permeability of molecules like mannitol—induce the release of lactate dehydrogenase from epithelial cells and cause distinct histological mucosal injury [85].

Furthermore, the majority of current research on permeation enhancers has focused on acute administration paradigms or short-term in vitro/in vivo models. Critical aspects including their long-term safety profile, intestinal barrier recovery capacity, and potential perturbations to immune homeostasis still lack systematic clinical validation. This renders permeation enhancers more suitable as adjunctive strategies for specific clinical scenarios, rather than as a universal solution for enhancing oral drug absorption [86]. In light of the aforementioned mechanistic limitations, this strategy requires strict constraints during its practical translation to clinical applications, and its implementation should also undergo comprehensive risk evaluation.

### 4.5. Nanotechnology

**Figure 4:**
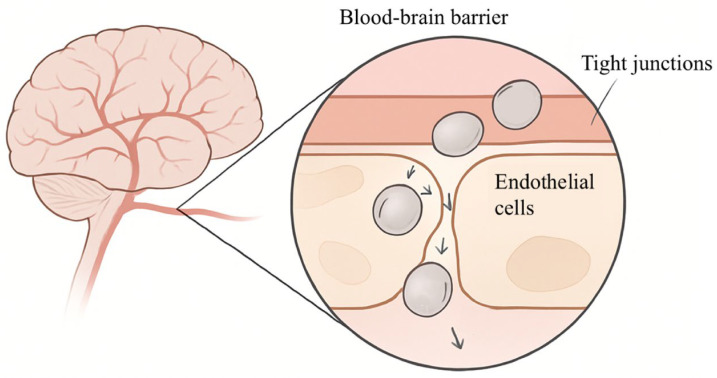
Schematic illustration of nanoparticle transport across highly restrictive biological barriers (using the BBB as an example).

In recent years, driven by the rapid advancement of nanotechnology, a growing number of pharmaceuticals have been delivered via nanocarriers. Nanotechnology, a drug delivery approach based on nanoscale particles, has been widely explored as a strategy to modulate drug transport across biological barriers, thereby offering potential solutions to permeability-limited drug delivery [87]. The blood–brain barrier (BBB) is one of the most selective and tightly regulated biological barriers in the human body and is frequently used as a representative model to illustrate the challenges associated with trans-barrier drug transport [88]. Research has demonstrated that over 95% of small-molecule agents and nearly all macromolecular substances fail to permeate the BBB effectively, highlighting the extreme permeability constraints imposed by highly restrictive biological barriers [89,90]. However, nanoparticles, by virtue of their small size and surface modifiability, have been investigated as potential tools to facilitate drug transport across highly restrictive biological barriers, with the BBB serving as a representative example. Certain nanocarriers have been reported to traverse highly restrictive barriers to a limited extent via paracellular pathways or endogenous transport mechanisms, observations that have been primarily derived from BBB-related studies and may provide insights into general trans-barrier transport behavior. Thus, the encapsulation of BCS III drugs into nanoparticles should be regarded as a highly context-dependent strategy for modulating drug–barrier interactions, rather than a universally applicable solution, with its effectiveness strongly influenced by the specific biological barrier involved. The transport of nanoparticles across highly restrictive biological barriers, exemplified by the BBB, is illustrated in Figure 4.

The primary categories of nanocarriers encompass lipid nanoparticles, polymeric nanoparticles, and nanocapsules, among others. Among these, polymeric nanocarriers have garnered significant attention in the field of nanodelivery systems, owing to their distinctive tunability and biodegradability [91]. Polymeric nanocarriers not only effectively enhance the solubility of drugs but also mitigate premature degradation, thereby improving drug stability. These carriers are broadly classified into natural and synthetic polymers: natural polymers such as chitosan, gelatin, and alginates typically exhibit low toxicity, favorable biocompatibility, and in vivo degradability; synthetic polymers like poly(lactic-co-glycolic acid) and polylactic acid can be precisely synthesized, enabling better control over drug release kinetics and conferring superior controlled-release and targeted delivery capabilities [92]. For example, Iqbal et al. constructed the composite polymeric nanoparticle PVP-HA loaded with metformin via electrospraying, using the synthetic polymer polyvinylpyrrolidone and natural polysaccharide hyaluronic acid as the matrix. This formulation notably enhanced the permeability of metformin and increased the AUC of the commercial metformin preparation by approximately 2.3-fold [93]. Madgulkar et al. enhanced the oral absorption of acyclovir via thiolated xyloglucan nanoparticulate technology, resulting in an approximately 2.575-fold increase in its AUC [94]. Hosny et al. improved the oral absorption of alendronate sodium using enteric-coated solid lipid nanoparticle technology, which enhanced its relative bioavailability by approximately 7.8-fold [95].

Furthermore, for the drug entity itself, the degradation rate is directly correlated with the type of carrier employed. Moreover, through structural engineering and surface modification, the nanosystem can be endowed with favorable controlled-release and sustained-release properties [96]. For example, Serrano-Mora et al. adsorbed ranitidine hydrochloride—a representative BCS III drug—encapsulated in solid lipid nanoparticles prepared via the thermal dispersion method onto the surface of directly compressible calcium hydrogen phosphate dihydrate. This modification prolonged the release of ranitidine (which otherwise exhibited nearly complete dissolution within 1 h) to a sustained 8 h profile. Furthermore, the similarity factor (f_2_) between the experimental release curve and the theoretical controlled-release curve reached 54.64, enabling oral controlled-release delivery that aligns with its pharmacokinetic properties [97].

While nanotechnology exhibits considerable potential for enhancing the bioavailability of BCS III drugs, it is not without limitations. For example, certain high-loading nanoformulations may undergo initial burst release or even dose dumping during the release phase, thereby posing potential safety hazard; Encapsulation efficiency and release modulation of BCS III drugs within lipid or polymeric carriers remain challenging; such issues can only be ameliorated by leveraging specialized nanostructures like mesoporous silica nanoparticles [98]; Given that the pore size of the normal BBB is merely ~4 nm, even nanoparticles struggle to traverse this barrier. In most cases, only a limited fraction of the drug can successfully cross the BBB, while concomitantly introducing the risk of off-target toxicity; Furthermore, the high complexity of nanostructures renders precise characterization of their architecture and behavior persistently challenging, thereby imposing stringent requirements on experimental methodologies and manufacturing processes. These challenges represent non-negligible risks in the development of nanoformulations, and addressing them is a prerequisite for the further advancement of nanotechnology-based oral delivery systems.

While nanotechnology has shown certain potential in enhancing the in vivo exposure of BCS III drugs, it still faces inherent mechanistic challenges. First, BCS III drugs are typically highly hydrophilic and strongly polar, whereas most nanocarriers depend on hydrophobic cores for drug encapsulation, leading to intrinsic incompatibility between the physicochemical properties of the drug molecules and the structural characteristics of the carriers [99]. Thus, in nanoformulations of BCS III drugs, low encapsulation efficiency, rapid drug leakage in the gastrointestinal environment, and initial burst release are prevalent phenomena. Under high drug loading conditions, such burst release may even trigger dose dumping, thereby introducing potential safety hazards [100]. For example, studies by Arpicco et al. have indicated that low-molecular-weight water-soluble drugs typically face issues of extremely low encapsulation efficiency and burst release when incorporated into liposomes or polymeric nanocarriers. To address this inherent incompatibility, researchers often need to employ complex strategies such as designing lipophilic prodrugs or hydrophobic ion pairs to artificially enhance the compatibility between the drug and the carrier core, thereby achieving more efficient encapsulation and improved stability [99].

Additionally, while some studies have observed the translocation of nanoparticles across the BBB in in vitro models or animal experiments, from the perspective of overall pharmacokinetics, the fraction of drug that actually reaches the brain tissue following oral administration remains extremely low [101]. For example, Ayub et al. have noted that while pegylated liposomes can extend the systemic circulation retention time of drugs, their accumulation in tissues such as the liver and spleen not only limits the effective delivery amount to the brain but also may induce severe systemic side effects. This tendency means that when nanosystems achieve central nervous system targeting, they often inevitably cause peripheral toxicity [102].

On the other hand, nanosystem architectures are highly complex, and their particle size distribution, surface properties, drug loading modalities, and in vivo dynamic behaviors are challenging to fully and accurately characterize. This not only introduces uncertainties into mechanistic investigations but also imposes more stringent demands on large-scale manufacturing, quality consistency control, and regulatory approval [103].

Thus, nanotechnology offers an important tool for the delivery of BCS III drugs, yet its utility is constrained by application contexts. Specifically for oral administration, this strategy is better suited as a supplementary measure to address issues of drug instability or fulfill specialized targeting demands.

### 4.6. SEDDS

SEDDS represent a formulation technology that spontaneously forms stable oil-in-water (O/W) emulsions or microemulsions upon contact with water or gastrointestinal fluids [104]. Upon spontaneous emulsification in vivo, these droplets form a stable nanoscale dispersion system for the drug; such a small-particle-size dispersion more effectively facilitates systemic absorption. This unique drug delivery system typically consists of an oil phase, surfactants, and a small amount of cosolvent. The oil phase plays a critical role in solubilizing the drug and promoting emulsification, while surfactants reduce surface tension to a certain extent, facilitating the formation of oil droplets and maintaining their stability. The cosolvent, in turn, functions to regulate the droplet size [105]. For example, Jakab et al. formulated baicalin into a SEDDS and observed that its in vitro reconstitution yielded a stable colloidal dispersion with a particle size of approximately 130–140 nm. This finding suggests that the combination of the three phases in an appropriate ratio can generate uniform emulsion droplets with small particle sizes [106]. Menzel et al. demonstrated that exenatide-loaded SEDDS, upon dilution, forms uniform emulsion droplets with a particle size of approximately 46 nm. This small particle size enhances the drug’s diffusion capacity in the mucus layer by approximately 3.7-fold and significantly improves its oral bioavailability [107]. Similarly, Djekic et al. leveraged this strategy to optimize the pharmacokinetic performance of acyclovir, a BCS III drug: the C_max_ was augmented by approximately 3.17-fold, while the T_max_ was shortened from 26 min to 14 min [108].

However, for highly hydrophilic BCS III drugs, direct solubilization in the oil phase and subsequent encapsulation into oil droplets are often challenging. This limitation can be notably mitigated by pre-complexing the drug with hydrophobic ion pairs. For example, Asad et al. formed hydrophobic ion pairs by combining an anionic surfactant with the cationic drug tobramycin (TOB). Their results showed that the logP value of TOB increased by approximately 1500-fold, accompanied by a zeta potential shift from positive to negative—indicating a substantial enhancement in TOB’s hydrophobicity [109].

Notably, as oil-phase-encapsulated dosage forms, both SEDDS and phospholipid complex strategies involve processes that mimic the absorption mechanism of natural lipids. Research has demonstrated that certain BCS III drugs may exhibit a negative food effect when co-administered with food. This phenomenon is primarily attributed to food-induced increases in gastrointestinal viscosity, which decelerate the disintegration and dissolution rates of the pharmaceutical formulation [31,110]. In contrast, lipid-based formulations can attenuate or even abolish the effect of food on oral bioavailability by recapitulating the intestinal lipid environment generated post-prandially. For example, Qian et al. demonstrated that when Probucol was incorporated into a lipid-based formulation, the plasma concentration-time profiles were nearly superimposable between fasting and postprandial administrations, thereby eliminating the pronounced food effect associated with the original formulation. This finding suggests that lipid-based formulations can mitigate or even negate the impact of differential food states on drug absorption [111].

Overall, within the absorption barrier framework, SEDDS function as a luminal environment-modulating strategy rather than a direct permeability-enhancing approach, capable of improving the oral bioavailability of BCS III drugs—particularly for compounds that exhibit partial compatibility with lipidic environments. However, their applicability remains constrained for highly hydrophilic drugs with insufficient affinity for the oil phase, often necessitating supplementary structural modification or pre-complexation strategies. Furthermore, the performance of SEDDS is closely linked to gastrointestinal physiological conditions, including bile secretion and food intake, which may introduce variability in in vivo absorption profiles.

### 4.7. Membrane Technology

Membrane technology refers to a class of techniques that involve fabricating or engineering thin films or multi-layered membranes as drug carriers or controlled-release architectures, with the aim of regulating drug release kinetics, targeting release sites, and enhancing drug stability [112]. By tailoring key physicochemical properties of membrane materials—including porosity, thickness, crosslinking density, and polymer hydrophilicity/hydrophobicity—precise modulation of drug release kinetics can be achieved [113]. Thin films are defined as single-layer membranes composed of a homogeneous material, primarily functioning to encapsulate, sustain-release, or stabilize drugs. Owing to their simplistic architecture, well-established fabrication processes, and cost-effectiveness, they have been extensively employed in pharmaceutical manufacturing. In contrast, composite membranes consist of two or more distinct materials, exhibiting more intricate structural configurations and versatile functional profiles. They are particularly suited for specialized drug delivery scenarios, such as those requiring pH responsiveness, enzymatic degradability, or mucoadhesive properties [114].

In contrast to alternative strategies, the flexible design and multifunctionality of membranes enable the rational tailoring of membrane materials to meet the specific requirements of different drugs. For example, Janardhanam et al. employed the layer-by-layer self-assembly technique to fabricate pH-responsive membranes via the alternating deposition of positively charged chitosan and negatively charged sodium alginate. Notably, the outer layer of these membranes was modified with folic acid-conjugated chitosan, thereby imparting targeted delivery capability. This design facilitates the precise targeting of colon cancer cells through the site-specific release of 5-fluorouracil (5-Fu) [115]; Tamilselvi et al. conjugated pectin with three distinct nucleotide moieties to successfully fabricate a 5-Fu-loaded composite membrane with colon-targeted delivery capability. This membrane facilitates the internalization of the drug into cancer cells via hydrogen bonding and electrostatic interactions, ultimately exerting an apoptotic effect on cancer cells [116].

Overall, membrane technology offers a highly versatile platform for modulating drug release kinetics and enabling targeted drug delivery via rational material and structural design. However, from the perspective of BCS III drug absorption, this strategy primarily addresses the site and rate of drug release rather than fundamentally overcoming the intrinsic trans-epithelial permeability barrier that limits oral bioavailability. Additionally, the in vivo performance of membrane-based systems is strongly influenced by gastrointestinal physiological variability—including pH fluctuations, enzymatic activity, and intestinal peristalsis—which may compromise the predictability of release behavior observed under controlled in vitro conditions [117]. For instance, Onoue et al. demonstrated that while certain controlled-release membrane systems exhibited consistent performance under controlled in vitro conditions, the drastic pH fluctuations in the highly dynamic gastrointestinal physiological environment directly altered the swelling and erosion kinetics of the polymer membranes, leading to significant deviations in drug release profiles [118]. Furthermore, the structural complexity of multilayer or composite membranes presents challenges for precise characterization, large-scale manufacturing, and quality consistency, thereby limiting their translational feasibility, particularly in large-scale manufacturing and long-term clinical use. Strategies for enhancing the delivery efficacy of BCS III drugs are summarized in Table 2.

### 4.8. Strategy Selection

**Figure 5:**
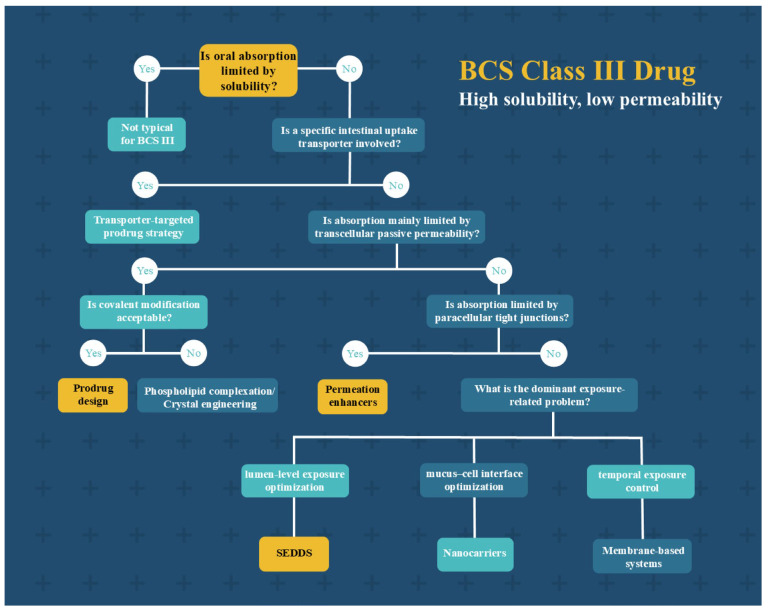
A mechanism-guided decision tree for oral absorption enhancement of BCS III drugs.

For BCS III drugs, the core limitation to oral absorption is intestinal permeability barriers rather than insufficient solubility. Therefore, when selecting absorption enhancement strategies, it is imperative to first identify the dominant rate-limiting step governing in vivo absorption, in conjunction with the physicochemical properties of the drug, thereby identifying the most appropriate modification approach. Based on the aforementioned mechanistic analyses, distinct absorption enhancement strategies exhibit substantial differences in their scope of application and translational potential. The mechanism-guided decision tree for improving oral absorption of BCS III class drugs is shown in Figure 5.

When the primary constraint of a drug arises from inadequate passive transcellular permeability, phospholipid complexation, prodrug design, and crystal engineering typically demonstrate high compatibility. Specifically, phospholipid complexation enhances absorption efficiency by improving the drug’s compatibility with biological membranes without altering its covalent structure; prodrug strategies can remodel transmembrane transport mechanisms at the molecular level, particularly for drugs with identifiable potential to utilize transporters; crystal engineering optimizes the balance between dissolution and permeation by regulating interfacial behaviors, offering favorable process controllability.

For BCS III drugs that are significantly limited by transporters or exhibit saturation effects during absorption, transporter-based prodrug strategies undoubtedly confer direct advantages. However, such approaches are highly sensitive to transporter expression levels and metabolic processes, and issues related to their stability and inter-individual variability must be thoroughly evaluated during the translational phase.

For drugs that are unstable in vivo or significantly affected by the gastrointestinal environment, SEDDS, nanotechnology, and membrane technologies can indirectly promote absorption by improving the mode of drug exposure in the intestine. Nevertheless, these strategies generally do not act directly on membrane permeability itself, and their translation and application are constrained by the complexity of their systems and safety concerns.

When drug absorption is primarily limited by the paracellular pathway, permeation enhancers can significantly increase drug flux in the short term, but their potential risk of disrupting the epithelial barrier limits their utility as a long-term oral strategy. Thus, such methods are more suitable as adjunctive or situational interventions rather than universal solutions.

From the perspectives of clinical translation and regulatory compliance, chemical modification currently stands as the most promising approach; phospholipid complexation and crystal engineering achieve a favorable balance between safety and absorption enhancement efficacy; while permeation enhancers, SEDDS, nano-delivery systems, and membrane technologies, despite demonstrating significant advantages at the experimental level, still face key bottlenecks in long-term safety and controllability that restrict their widespread application. Therefore, absorption enhancement for BCS III drugs should be based on mechanism-guided strategy selection rather than the generalized application of a single technology. This provides a theoretical foundation for the subsequent development of a systematic decision-making framework and guidance for practical formulation development.

## 5. Future Outlook

### 5.1. Mechanism-Based Classification-Guided Drug Design

Although various molecular modification and drug delivery strategies have been developed in recent years to address the limited oral absorption of BCS III drugs, there is still a lack of a universal technology that can significantly enhance the oral bioavailability of all BCS III drugs. This phenomenon is not due to the insufficiency of a single technical approach, but mainly because of the high heterogeneity in the intestinal absorption mechanisms of BCS III drugs. Different drugs may be limited by different rate-limiting steps during oral absorption, such as insufficient passive transcellular permeability, restricted transporter-mediated processes, blocked paracellular pathways, or poor in vivo stability. The coexistence of multi-level and multi-mechanism absorption barriers determines that any single strategy is difficult to achieve consistent and predictable absorption enhancement effects across different drugs. Therefore, future research should shift the focus forward, classifying BCS III drugs based on intestinal permeability barriers before formulating or molecular design, and matching corresponding drug design or drug delivery strategies accordingly to improve the specificity and overall efficiency of the research.

### 5.2. Clinical Translation of Prodrug Strategies

Based on the comparative analysis presented above, chemical modification strategies—particularly transporter-targeted prodrug design—are recognized as one of the most clinically translatable approaches for enhancing oral absorption to date. However, even these high-potential strategies are subject to substantial limitations in their applicability due to multiple intrinsic factors. The transporter-mediated absorption process is inherently highly complex: its efficacy depends not only on the structural properties of the drug molecule but also correlates closely with intestinal transporter expression levels, saturation kinetics, and inter-individual physiological variability [119].

This multi-factorial regulatory mechanism often results in prodrugs that exhibit favorable absorption profiles in in vitro models yet display markedly divergent absorption behaviors under in vivo conditions, thereby compromising the predictability of their absorption efficacy. Future research should prioritize elucidating the systematic relationships between drug structure, transporter affinity, and in vivo absorption performance to improve the stability and reliability of prodrug strategies during clinical translation.

### 5.3. Reassessment of Barrier Modulation Strategies

In contrast to molecular modification strategies, permeation enhancers, nanodelivery systems, and membrane-modulation technologies typically regulate the intestinal epithelial barrier or local exposure microenvironment through non-specific mechanisms. While these approaches can significantly increase drug flux across the intestinal barrier under specific experimental conditions, their mechanisms of action often involve perturbation of intestinal barrier integrity—leading to substantial uncertainties regarding long-term safety, effect controllability, and regulatory acceptability.

Owing to these concerns, such strategies face notable limitations as universal oral absorption enhancers. Future research, while continuing to explore their absorption-enhancing potential, should prioritize more rigorous assessments of their safety thresholds and applicability boundaries from the perspectives of mechanistic rationality and clinical feasibility. Strategy selection should not be based solely on absorption improvement observed under short-term experimental conditions [62].

## 6. Conclusions

This review systematically compares the major strategies developed in recent years for enhancing the oral absorption of BCS III drugs, and analyzes their respective advantages and limitations from the dual perspectives of mechanism of action and translational feasibility. The analysis reveals that the limited oral absorption of BCS III drugs does not stem from inadequate formulation optimization, but is predominantly governed by the highly complex and tightly regulated intrinsic mechanisms underlying intestinal permeability. This characteristic dictates the substantial disparities in the applicability and actual efficacy of different strategies.

Comprehensive comparative findings indicate that strategies targeting molecular or transport mechanisms theoretically exhibit higher specificity; however, their practical efficacy is frequently constrained by mechanistic complexity, transporter saturation, and physiological variability, rendering it challenging to sustain consistent effectiveness across diverse drugs and populations. In contrast, strategies relying on barrier modulation or drug delivery system optimization, while demonstrating certain absorption-enhancing potential under experimental conditions, continue to confront fundamental challenges regarding safety, controllability, and clinical translatability. These mechanistic-level discrepancies collectively suggest that no universal oral absorption enhancement regimen exists that can be broadly applied to all BCS III drugs.

Accordingly, the key advancement in future research will not lie in the continuous expansion of the number of strategies, but rather in clarifying the applicable boundaries of different strategies through mechanism-guided comparative studies, and fully integrating considerations of physiological relevance, safety, and individual variability at the early stages of formulation design—thereby reducing uncertainties in the translation of experimental research to clinical practice. The analytical framework proposed in this review provides a foundation for the rational design and strategic selection of oral formulations for BCS III drugs.

## Figures and Tables

**Table 1:** BCS Classification System.

Category	Solubility	Permeability	Characteristics	Representative Drugs
Class I	High	High	Oral absorption is usually complete.	Diazepam, Propranolol, Paracetamol
Class II	Low	High	Oral absorption is limited by dissolution.	Ibuprofen, Ketoprofen, Fluconazole
Class III	High	Low	Oral absorption is limited by membrane permeability.	Captopril, Amikacin, Acyclovir
Class IV	Low	Low	Shows the poorest oral absorption among BCS classes.	Erythromycin, Rifampicin, Cyclosporine A

**Table 2:** Strategies for Enhancing BCS III Drugs.

Strategy	Mechanism	Advantages	Limitations	Magnitude of Enhancement
Phospholipid Complexation Strategy	Upon complexation with phospholipids, the drug conceals polar hydrogen bonds, mimics the lipid bilayer structure, mitigates P-gp-mediated efflux, and enhances membrane permeability.	Excellent biocompatibility; simultaneous enhancement of solubility and permeability; attenuation of efflux transporter-mediated efflux.	The physicochemical properties of the complex are insufficiently stable; the preparation process is intricate with limited characterization approaches; there exists a risk of batch-to-batch variability	The AUC of FEX was increased by approximately 3.38-fold [44]; The AUC of bergenin was increased by approximately 4.39-fold, and the C_max_ was increased by approximately 4.46-fold [45]; The bioavailability of the salvianolic acid B phospholipid complex was enhanced by approximately 2.3-fold [46].
Prodrug	Lipophilicity enhancement or introduction of transporter substrate moieties via esterification and other strategies enables the release of the parent drug in vivo through enzymatic or chemical reactions.	Can significantly enhance permeability and oral bioavailability (BA); enables active absorption via transporters; mitigates saturated absorption and prolongs drug absorption duration.	Dependence on in vivo metabolic transformation with substantial inter-individual variability; potential formation of toxic metabolites; stringent development and regulatory requirements	The AUC of gabapentin enacarbil was increased by 1.68-fold [59,60]; The AUC of C2E5 was elevated to a level comparable to that observed following intravenous administration [61]; Increase the oral bioavailability of gabapentin acetylcarbimab to over 68% [62].
Crystal Engineering	Through modulation of crystal packing and intermolecular interactions, the dissolution driving force and membrane partitioning behavior are enhanced, consequently promoting transmembrane diffusion.	Flexible modulation of the solubility-permeability balance; stable solid-state morphology facilitating industrial-scale manufacturing; applicability to salt-resistant molecules.	There is a risk of SMPT, which may offset the advantages; it is necessary to have a systematic connection between dissolution and phase transition characterization; the workload of early screening in the development stage is large.	The 8 h cumulative permeation of famotidine was enhanced by approximately 1.75-fold through salt-cocrystallization [70]; The AUC of biochanin A was increased by approximately 2.1-fold via cocrystallization with nicotinamide [71]; The AUC of the 5-fluorouracil–2,5-dihydroxybenzoic acid cocrystal (5FU-25DHBA) was enhanced to 2.78-fold that of the parent drug [72].
Permeation Enhancers	Epithelial permeability can be transiently enhanced via solvent drag or modulation of membrane architecture.	Rapid onset of action with broad applicability to hydrophilic drugs; marked enhancement of transmembrane permeation for small-molecule hydrophilic agents; relatively simple formulation.	Potential impairment of the epithelial barrier; narrow therapeutic safety window; unclear risks associated with long-term or high-dose administration; susceptibility to variations in dosing conditions.	The bioavailability of ranitidine, atenolol, and acyclovir was enhanced by 1.68-fold, 1.67-fold, and 2.32-fold, respectively [78]; The AUC of acyclovir was enhanced by 10.09-fold [79].
Nanotechnology	Nano-carrier-based delivery systems enhance drug solubility, afford protection to the drug payload, improve mucosal permeability, and augment targeting specificity.	Enables solubility enhancement, controlled/sustained release, and targeted delivery; allows partial penetration of barriers such as the BBB; and reduces premature degradation.	Potential risk of dose dumping; challenges in achieving satisfactory encapsulation efficiency and precise release regulation; particle size remains constrained by the pore size of the BBB; complex characterization processes.	The AUC of metformin was enhanced by 2.3-fold [93]; The AUC of Acyclovir was enhanced by approximately 2.575-fold [94]; The relative bioavailability of alendronate sodium was enhanced by approximately 7.8-fold [95].
SEDDS	Composed of an oil phase, surfactant, and cosolvent, it self-assembles into nanoemulsion droplets upon entering the gastrointestinal tract, thereby enhancing drug solubility and diffusion across the mucus layer.	The formation of 10–100 nm nanoemulsion droplets expands the contact area; additionally, it mitigates negative food effects and exhibits a high drug-loading capacity.	Hydrophilic BCS III drugs require prior formation of hydrophobic ion pairs; the formulation is complex with high optimization difficulty; certain surfactants have safety limits	The C_max_ of acyclovir was increased by approximately 3.17-fold, and the time to reach T_max_ was reduced from 26 min to 14 min [108]; The logP of TOB was increased by approximately 1500-fold [109].
Membrane-based Technologies	Thin films or multilayer films enable the spatiotemporal control of drug release rate and site, thereby achieving gastrointestinal segment-specific or colon-targeted sustained release.	High structural designability; enables pH-, enzyme-, and ligand-responsive behaviors; facilitates localized high drug concentrations while effectively mitigating systemic toxicity; relatively mature manufacturing processes.	Limited drug-loading capacity; insufficient biodegradability of partial materials; susceptibility to burst release; scalability of manufacturing and stability profiles require further optimization.	Enables unilateral directional release of 5-FU with mucosal adhesion, thereby enhancing colonic permeability and the inhibitory efficacy against cancer cells [115]; Under the modulation of the bio-composite membrane, 5-FU exhibits slower and more sustained drug release, with enhanced cellular uptake by colon cancer cells and potentiated apoptosis-inducing efficacy [116].

## Data Availability

No new data were created or analyzed in this study. Data sharing is not applicable to this article.

## Abbreviations

The following abbreviations are used in this manuscript:

P-gp	P-glycoprotein
SEDDS	Self-Emulsifying Drug Delivery System
SMPT	Solution-mediated phase transformation
BBB	Blood–brain barrier
TJs	Tight junctions
